# Prognostic Impact of Cumulative Fluid Balance in Critically Ill Patients: A Retrospective Cohort Study

**DOI:** 10.7759/cureus.104023

**Published:** 2026-02-21

**Authors:** Eugeniu Gisca, Simone Costa, Marta Magno, Catia R Santos, Ana Araújo

**Affiliations:** 1 Critical Care Medicine, Unidade Local de Saúde da Região de Leiria - Hospital de Santo André, Leiria, PRT

**Keywords:** critically ill patients, cumulative fluid balance (cfb), icu patients, negative fluid balance, positive fluid balance

## Abstract

Background

Cumulative fluid balance (CFB) reflects net fluid accumulation during intensive care unit (ICU) stay. However, prognostically relevant thresholds at ICU discharge remain insufficiently characterised. This study aimed to evaluate the association between CFB measured at ICU discharge and clinical outcomes in critically ill patients.

Methods

We conducted a retrospective single-centre cohort study including adult critically ill patients admitted to a tertiary mixed ICU between September 2022 and September 2023. CFB was calculated as the net difference between total fluid input and output during ICU stay and expressed as a percentage of baseline body weight at ICU discharge. Predefined CFB thresholds (>7%, −3% to 7%, and <−3%) were selected to reflect clinically relevant degrees of fluid accumulation and depletion. The primary outcome was ICU mortality. Secondary outcomes included the requirement for FiO₂ >0.60, duration of invasive mechanical ventilation, increase in serum creatinine >50% from baseline, need for renal replacement therapy (RRT), and length of stay.

Results

A total of 93 patients were analysed. ICU mortality differed significantly across CFB groups, being highest in patients with CFB >7% (n=10, 33.3%) compared with −3% to 7% (n=5, 14.3%) and <−3% (n=2, 7.1%) (p = 0.027). Requirement for FiO₂ >0.60 was more frequent in the CFB >7% group (n=13, 43.3%; p = 0.007), and median duration of mechanical ventilation was longer in this group (11 days, IQR 6.1-12.3; p = 0.029). Patients with CFB <−3% demonstrated numerically higher rates of creatinine increase >50% (n=8, 28.6%) and RRT (n=5, 17.9%), although these differences were not statistically significant. Hospital mortality and length of stay did not differ significantly between groups. Baseline severity scores were comparable across groups.

Conclusions

CFB at ICU discharge was associated with distinct prognostic patterns in critically ill patients. A markedly positive CFB (>7%) was associated with higher ICU mortality and greater respiratory support requirements, whereas a markedly negative CFB (<−3%) showed a trend towards less favourable renal outcomes. These findings suggest that CFB may serve as a complementary prognostic marker in the later phases of critical illness, although causal inferences cannot be established from this retrospective analysis.

## Introduction

Fluid therapy is a cornerstone of critical care management, yet its optimal application remains controversial. Intravenous fluid administration evolves through distinct clinical phases: initial resuscitation to restore organ perfusion, subsequent titration to maintain adequate tissue perfusion, and eventual de-escalation through minimisation of fluid administration and mobilisation of accumulated fluid. Given the heterogeneity of critically ill patients in terms of admission source, underlying pathophysiology, and illness trajectory, fluid management remains inherently complex. No universally accepted method reliably identifies patients who will benefit from additional fluid administration [1].

Observational studies consistently report associations between cumulative fluid balance (CFB) and adverse outcomes, including mortality, acute kidney injury (AKI), and prolonged mechanical ventilation [1]. Positive CFB has been associated with increased mortality in patients with sepsis [2-4], AKI [5], and surgical populations [6]. A systematic review of observational studies further confirmed that both fluid overload and positive CFB are associated with increased mortality in critically ill patients [7]. However, interpretation is limited by heterogeneity in definitions, timing, and methods used to quantify CFB. Similar relationships between CFB and adverse outcomes have also been described in highly selected populations, such as patients with acute respiratory distress syndrome receiving extracorporeal membrane oxygenation [8]. Moreover, critically ill patients demonstrate variable fluid balance trajectories, with only a minority conforming to the idealised four-phase model of fluid therapy [9].

Terminological imprecision further complicates interpretation. The term "fluid overload" is often applied broadly to positive fluid balance, although not all positive CFB is necessarily harmful [10], and reports suggest that both excessive positive and negative CFB may be associated with unfavourable outcomes [9]. In addition, no universally applicable prognostic threshold has been established, with reported cut-offs varying across populations [10]. Estimation of CFB based on fluid input-output charts is also subject to measurement inaccuracies and should not be considered a direct surrogate of true body fluid status [11].

While prior studies have largely focused on fluid balance during early phases of critical illness [1], less attention has been given to CFB at ICU discharge - a clinically meaningful timepoint reflecting the cumulative effect of fluid strategies throughout the ICU stay. The prognostic implications of CFB assessed specifically at ICU discharge remain insufficiently characterised.

Therefore, this study aimed to evaluate the association between CFB at ICU discharge and clinical outcomes in critically ill patients.

## Materials and methods

Trial design and setting

We conducted a retrospective, single-centre cohort study in a tertiary polyvalent adult ICU. All patients admitted consecutively to the ICU between September 2022 and September 2023 were screened for eligibility. During the study period, a total of 318 patients were admitted. Of these, 225 patients were excluded for meeting at least one exclusion criterion, and 93 patients were included in the final analysis. The inclusion and exclusion criteria are summarised in Table 1.

**Table 1:** Inclusion and exclusion criteria

Inclusion criteria	Exclusion criteria
Age ≥ 18 years	ICU length of stay < 24 hours or death within the first 24 hours after ICU admission
Requirement for level 3 critical care (≥ 2 organ dysfunctions)	Pregnancy or postpartum status
Complete and correctly recorded clinical data, including fluid balance and outcome variables	Single-organ dysfunction requiring level 2 care only
-	Admission for donor maintenance care (potential organ donors)
-	Failure to meet all inclusion criteria

Owing to the retrospective nature of the study and the use of anonymised routinely collected clinical data, the requirement for informed consent was waived according to institutional policy.

Outcomes

The primary outcome was ICU mortality. Secondary outcomes included the requirement for fractional inspired oxygen (FiO₂) greater than 0.60 during ICU stay, total duration of invasive mechanical ventilation, renal function deterioration defined as an increase in serum creatinine greater than 50% from baseline, and the requirement for renal replacement therapy (RRT) at any point during ICU length of stay. ICU and hospital mortality, as well as ICU and hospital length of stay, were also recorded. Outcomes were assessed at ICU discharge or death.

Data sources

Clinical data were extracted retrospectively from the ICU clinical information systems B.ICU.Care and the hospital electronic medical record (SClínico Hospitalar). Collected variables included demographic characteristics, admission diagnosis, major comorbidities, and routinely recorded clinical and laboratory data. Severity of illness was assessed using the Sequential Organ Failure Assessment (SOFA) score, Acute Physiology and Chronic Health Evaluation II (APACHE II) score, and Simplified Acute Physiology Score II (SAPS II).

Fluid intake and output data were obtained from nursing documentation in the B.ICU.Care system and included all intravenous fluids, enteral and parenteral nutrition, blood products, urine output, drain losses, and ultrafiltration volumes in patients receiving RRT. Nevertheless, undocumented sources of fluid administration ("fluid creep") may not have been fully captured in intake-output records.

CFB was calculated as the net difference between total fluid input and total fluid output during ICU stay and expressed as a percentage of baseline body weight. CFB was assessed at the time of ICU discharge. Patients were stratified into three groups according to CFB: >7%, −3% to 7%, and <−3%. These thresholds were predefined to represent clinically relevant degrees of fluid accumulation and depletion and were informed by prior observational literature describing adverse outcomes at similar ranges of CFB.

Statistical analysis

Statistical analysis was performed using IBM SPSS Statistics for Windows, Version 20 (Released 2011; IBM Corp., Armonk, New York). Continuous baseline characteristics were checked for normality using the Shapiro-Wilk test. No variables were found to have a normal distribution; therefore, all continuous variables were reported as medians with interquartile ranges (IQRs). Categorical variables were reported as a number (percentage).

Comparisons between the three CFB groups were carried out using the Kruskal-Wallis rank sum test for continuous variables and the chi-squared test for categorical variables. Differences were considered statistically significant when the p-value was less than 0.05.

To further explore the association between the CFB group and ICU mortality, a multivariable logistic regression model was constructed with ICU mortality as the dependent variable and the CFB group as the independent variable of interest, adjusting for baseline illness severity using the APACHE II score. Adjusted odds ratios (ORs) with 95% confidence intervals (CIs) were calculated.

## Results

Study population

During the study period, a total of 318 patients were admitted to the ICU. Of these, 225 patients were excluded for not meeting the predefined inclusion and exclusion criteria, resulting in 93 patients included in the final analysis. Patients were stratified according to CFB at ICU discharge into three groups: CFB >7% (n=30), CFB −3% to 7% (n=35), and CFB <−3% (n=28).

Baseline characteristics

The most common primary admission diagnosis was community-acquired pneumonia, accounting for 24.7% (n=23) of cases, followed by intra-abdominal infection (20.4%, n=19). Nosocomial pneumonia, thoracic or abdominal trauma, and post-cardiac arrest syndrome were each observed in 15.1% of patients [14]. Urosepsis represented 9.7% (n=9) of ICU admissions in the study cohort, reflecting a heterogeneous critically ill population. Baseline demographic characteristics, comorbidities, and severity of illness scores are summarised in Table 2.

**Table 2:** Baseline characteristics of the study population according to cumulative fluid balance (CFB) at ICU discharge Values are reported as median (interquartile range) or number (percentage). P-values refer to comparisons across the three CFB groups. CKD – chronic kidney disease; DM – diabetes mellitus; SOFA – Sequential Organ Failure Assessment; APACHE II – Acute Physiology and Chronic Health Evaluation II; SAPS II – Simplified Acute Physiology Score II.

Characteristic	CFB −3% to 7% (n=35)	CFB < −3% (n=28)	CFB > 7% (n=30)	Total (n=93)	p-value
Age, years	71.0 (64.0–77.0)	72.0 (64.8–78.2)	73.5 (68.2–78.5)	72.0 (65.0–78.0)	0.503
Male sex, n (%)	11 (31.4)	14 (50.0)	13 (43.3)	38 (40.9)	0.311
SOFA score	11.0 (10.0–13.0)	12.0 (8.8–13.0)	10.5 (9.0–12.0)	11.0 (9.0–13.0)	0.653
APACHE II score	18.0 (18.0–19.0)	19.0 (18.0–20.5)	18.0 (18.0–20.0)	18.0 (18.0–20.0)	0.195
SAPS II score	31.0 (27.5–39.5)	33.0 (30.0–60.0)	31.0 (30.0–45.0)	32.0 (29.0–47.0)	0.180
DM, n (%)	7 (20.0)	6 (21.4)	13 (43.3)	26 (28.0)	0.073
Hypertension, n (%)	19 (54.3)	16 (57.1)	11 (36.7)	46 (49.5)	0.228
Obesity, n (%)	8 (22.9)	7 (25.0)	11 (36.7)	26 (28.0)	0.426
CKD, n (%)	1 (2.9)	3 (10.7)	2 (6.7)	6 (6.5)	0.450

The median age of the overall cohort was 72 years (IQR 65-78), and 59.1% (n=55) of patients were female. At ICU admission, the median SOFA score was 11 (IQR 9-13), the median APACHE II score was 18 (IQR 18-20), and the median SAPS II score was 32 (IQR 29-47), indicating a population with substantial critical illness severity.

Hypertension was the most prevalent comorbidity, affecting 49.5% (n=46) of patients, followed by type 2 diabetes mellitus and obesity, each present in 28.0% (n=26) of the cohort. Pre-existing chronic kidney disease (CKD) greater than stage II was identified in 6.5% (n=6) of patients.

Baseline demographic characteristics, severity of illness scores, and major comorbidities were comparable across the three CFB groups, with no statistically significant differences observed, indicating relatively homogeneous groups and a similar severity of illness at ICU admission.

Primary outcome

ICU mortality differed significantly across CFB categories (p = 0.027, χ²(2) = 7.25, Cramér’s V = 0.28). Patients with CFB >7% had the highest ICU mortality rate (33.3%, n=10), compared with 14.3% (n=5) in the CFB −3% to 7% group and 7.1% (n=2) in the CFB <−3% group (Table 3). In multivariable logistic regression analysis adjusted for APACHE II score, patients with CFB >7% demonstrated higher odds of ICU mortality compared with the reference group (−3% to 7%) (adjusted OR 3.02, 95% CI 0.90-10.17; p=0.075). No significant association was observed for CFB <−3% (adjusted OR 0.49, 95% CI 0.09-2.76; p=0.415). The APACHE II score was not independently associated with ICU mortality in this model.

**Table 3:** Clinical outcomes according to cumulative fluid balance (CFB) at ICU discharge Values are reported as median (interquartile range) or number (percentage). P-values refer to comparisons across the three CFB groups. Cr – creatinine; LOS – length of stay; MV – invasive mechanical ventilation; RRT – renal replacement therapy.

Outcome	CFB −3% to 7%	CFB < −3%	CFB > 7%	Total (n=93)	p-value
ICU mortality, n (%)	5 (14.3)	2 (7.1)	10 (33.3)	17 (18.3)	0.027
Hospital mortality, n (%)	9 (25.7)	6 (21.4)	13 (43.3)	28 (30.1)	0.148
FiO₂ > 0.60, n (%)	6 (17.1)	3 (10.7)	13 (43.3)	22 (23.7)	0.007
MV, days	8.2 (6.2–9.9)	7.9 (6.3–9.1)	11.0 (6.1–12.3)	8.9 (6.2–10.8)	0.029
Cr increase >50%, n (%)	6 (17.1)	8 (28.6)	2 (6.7)	16 (17.2)	0.087
RRT, n (%)	2 (5.7)	5 (17.9)	2 (6.7)	9 (9.7)	0.214
ICU LOS, days	6.0 (4.5–8.5)	7.0 (5.0–9.0)	6.0 (5.0–8.0)	6.0 (5.0–8.5)	0.271
Hospital LOS, days	17.0 (13.5–22.0)	19.0 (15.0–24.2)	17.0 (14.2–21.0)	18.0 (14.0–22.0)	0.320

Secondary outcomes

The requirement for FiO₂ greater than 0.60 during ICU stay was significantly more frequent in patients with CFB >7% (43.3%, n=13) compared with those with CFB −3% to 7% (17.1%, n=6) and CFB <−3% (10.7%, n=3) (p = 0.007, χ²(2) = 9.85, Cramér’s V = 0.33).

The duration of invasive mechanical ventilation differed significantly between groups (p = 0.029), with patients in the CFB >7% group exhibiting a longer median duration of mechanical ventilation (11.0 days, IQR 6.1-12.3) compared with the other two groups.

Renal outcomes demonstrated a different pattern. An increase in serum creatinine greater than 50% from baseline occurred more frequently in patients with CFB < −3% (28.6%, n=8) compared with those with CFB −3% to 7% (17.1%, n=6) and CFB >7% (6.7%, n=2), although this difference did not reach statistical significance (p = 0.087, χ²(2) = 4.88, Cramér’s V = 0.23). Similarly, the requirement for RRT was higher in the CFB <−3% group (17.9%, n=5), but this difference was not statistically significant (p = 0.214, χ²(2) = 3.08, Cramér’s V = 0.18). Given the limited number of renal events, these findings should be interpreted as exploratory and hypothesis-generating rather than definitive.

Hospital mortality differed numerically across CFB groups without reaching statistical significance. Patients in the CFB >7% group exhibited the highest hospital mortality rate (43.3%, n=13) (p = 0.148, χ²(2) = 3.82, Cramér’s V = 0.20).

Length of stay outcomes also showed numerical differences between groups. Both ICU length of stay (7 days, IQR 5-9) and hospital length of stay (19 days, IQR 15.0-24.2) appeared higher in patients with CFB < −3%, although these differences were not statistically significant (ICU length of stay: p = 0.271; hospital length of stay: p = 0.320).

## Discussion

This single-centre retrospective cohort study evaluated the association between CFB at ICU discharge and clinical outcomes in critically ill patients. Patients with a CFB >7% demonstrated higher ICU mortality and greater respiratory support requirements (higher FiO₂ and a longer duration of invasive mechanical ventilation) compared with those with neutral or negative balances, whereas patients with a markedly negative CFB (<−3%) showed numerically less favourable renal outcomes. Baseline severity scores were comparable across groups, and adjustment for the APACHE II score did not materially alter the direction of association, although statistical significance was attenuated (adjusted OR 3.02, 95% CI 0.90-10.17). Given the limited sample size and wide CIs, these findings should be interpreted cautiously. Importantly, CFB may represent a marker of illness severity or haemodynamic instability rather than a direct causal mediator of adverse outcomes, and the study cannot determine whether fluid accumulation is intrinsically harmful or simply reflects greater clinical complexity.

The association between positive fluid balance and adverse outcomes observed in this study is consistent with prior observational evidence. Multiple studies have demonstrated an increased risk of mortality associated with positive CFB in heterogeneous critically ill populations, including patients with sepsis, AKI, and those undergoing major surgery [7,12]. Earlier landmark studies on septic shock similarly reported independent associations between positive fluid balance, elevated central venous pressure, and increased mortality [13]. In other septic cohorts, persistent positive fluid balance during later ICU days has been associated with increased long-term mortality [1], while in postoperative cardiac surgery patients, early achievement of negative fluid balance has been linked to improved survival [6]. Furthermore, positive fluid balance at ICU discharge has previously been identified as an independent predictor of post-discharge mortality [1]. The present study extends this body of evidence by focusing specifically on CFB assessed at ICU discharge, rather than during earlier resuscitation or stabilisation phases, a timepoint that remains comparatively underexplored in the literature.

The significantly higher requirement for supplemental oxygen and prolonged mechanical ventilation in patients with CFB >7% aligns with previous observations linking fluid accumulation to respiratory dysfunction. Excess interstitial and extravascular lung water may impair gas exchange and lung compliance, thereby increasing ventilatory support requirements [14]. Contemporary studies emphasise the importance of fluid balance trajectories, rather than isolated absolute values, in determining respiratory and haemodynamic consequences [9]. This dynamic perspective may partly explain why static CFB measurements at single timepoints incompletely capture the complex relationship between fluid management and organ-specific outcomes.

Renal outcomes in the present study followed a different pattern from respiratory and mortality endpoints. Patients with CFB <−3% demonstrated a numerically higher incidence of significant creatinine increase and requirement for RRT, although these findings did not reach statistical significance. Fluid accumulation has also been strongly associated with impaired renal recovery and increased mortality among critically ill patients with AKI [15]. Similar observations have been reported in studies exploring fluid balance trajectories, suggesting that both excessive positive accumulation and excessive negative depletion may be associated with unfavourable outcomes, depending on the timing and clinical context [9,16]. In the present cohort, the numerically higher incidence of renal dysfunction and longer ICU and hospital length of stay, observed in patients with markedly negative CFB, may reflect greater clinical complexity or challenges in achieving optimal fluid management, rather than a direct deleterious effect of negative balance itself. However, given the limited number of renal events, these observations should be interpreted cautiously.

Several conceptual and methodological considerations are relevant when interpreting these findings. First, positive CFB should not be simplistically equated with true fluid overload, as intravascular volume status and interstitial fluid accumulation may diverge substantially in critically ill patients [10]. Second, no universally accepted CFB threshold reliably predicts adverse outcomes across heterogeneous ICU populations [10]. Third, estimation of fluid balance based on intake-output records is inherently imprecise, particularly over prolonged ICU stays, as insensible losses and cumulative documentation errors may substantially affect accuracy [11]. The clinical implications of assessing CFB at ICU discharge, rather than during earlier phases of critical illness, remain incompletely characterised and warrant further investigation in larger prospective cohorts.

From a clinical perspective, these findings highlight the limitations of uniform fluid management strategies. The observed association between CFB >7% and adverse respiratory outcomes and ICU mortality raises the hypothesis that avoidance of excessive fluid accumulation during later phases of critical illness may warrant consideration. Furthermore, active deresuscitation strategies aimed at removing iatrogenic fluid overload have been associated with reduced mortality in critically ill patients [17]. However, the potential signal of increased renal morbidity in patients with markedly negative CFB indicates that aggressive fluid de-escalation should be approached cautiously and individualised according to physiological markers of perfusion and organ function. Increasing evidence suggests that fluid balance trajectories, rather than absolute cumulative values alone, may provide a more informative framework for guiding fluid stewardship in the ICU [9].

Future prospective studies incorporating serial fluid balance trajectories and dynamic haemodynamic assessment are warranted to better define causality and optimal fluid stewardship strategies.

Limitations

This study has several limitations. Its retrospective design precludes causal inference, and observed associations may reflect residual confounding despite adjustment for baseline severity using the APACHE II score. Although multivariable logistic regression was performed, adjustment was limited to the APACHE II score due to the relatively small number of mortality events. This constrained the robustness and comprehensiveness of the model and resulted in wide CIs and potential instability. Residual confounding and model instability, therefore, cannot be excluded.

The single-centre setting limits external generalisability, particularly to ICUs with different case mixes or fluid management practices. Additionally, CFB was derived from routinely documented intake-output records, which may be subject to recording inaccuracies, cumulative documentation errors, and a failure to capture insensible losses. As such, intake-output-based estimates may under- or overestimate true fluid status compared with weight-based methods and do not necessarily reflect intravascular or total body fluid distribution [11]. Unmeasured factors, including fluid type, timing of administration, vasoactive support, and underlying sepsis status, may have influenced outcomes independently of CFB.

Importantly, CFB was assessed at ICU discharge, which introduces the potential for survivor bias. Patients who died early during ICU stay may have exhibited different fluid balance trajectories that were not captured in discharge-based assessment. This temporal limitation may influence the interpretation of the observed associations and limit causal inference regarding fluid balance trajectories.

Finally, the exclusive inclusion of patients requiring level 3 care limits extrapolation of these findings to less severely ill ICU populations.

## Conclusions

This retrospective cohort study found that positive CFB at ICU discharge was associated with higher ICU mortality and greater respiratory support requirements, whereas markedly negative fluid balance showed unfavourable renal trends. These findings contribute to the growing body of observational evidence suggesting that extremes of fluid balance may be associated with adverse outcomes in critically ill patients. However, given the study’s retrospective design and limited sample size, these results should be considered hypothesis-generating. Prospective studies evaluating longitudinal fluid balance trajectories and their relationship with dynamic markers of tissue perfusion and organ function are warranted to better clarify causality and potential clinical implications.
